# Frequency and characteristics of bacterial and viral low-grade infections of the intervertebral discs: a prospective, observational study

**DOI:** 10.1186/s10195-022-00633-y

**Published:** 2022-03-18

**Authors:** Wolfgang Senker, Stefan Aspalter, Christian Radl, Josef Pichler, Stefan Doppler, Serge Weis, Christine Webersinke, Helga Wagner, Philipp Hermann, Martin Aichholzer, Kathrin Aufschnaiter-Hießböck, Wolfgang Thomae, Nico Stroh, Thomas Hauser, Andreas Gruber

**Affiliations:** 1 Department of Neurosurgery, Kepler University Hospital, Johannes Kepler University, Wagner-Jauregg Weg 15, 4020 Linz, Austria; 2 Department of Internal Medicine, Kepler University Hospital, Johannes Kepler University, Wagner-Jauregg Weg 15, 4020 Linz, Austria; 3 Institute of Pathology and Microbiology, Kepler University Hospital, Johannes Kepler University, Krankenhausstraße 9, 4021 Linz, Austria; 4 Institute of Pathology and Neuropathology, Kepler University Hospital, Johannes Kepler University, Wagner-Jauregg Weg 15, 4020 Linz, Austria; 5 Center for Clinical Studies (CCS Linz), Johannes Kepler University, Huemerstrasse 3-5, 4020 Linz, Austria; 6 Department of Medical Statistics and Biometry, Institute of Applied Statistics, Johannes Kepler University, Altenberger Strasse 69, 4040 Linz, Austria

**Keywords:** Low back pain, Bacterial colonization, Intervertebral discs, Low-grade infection, Modic changes, *Cutibacterium acnes*, Coagulase-negative staphylococci, Herpes simplex

## Abstract

**Study design:**

Monocentric, prospective, observational study.

**Objective:**

The clinical relevance of bacterial colonization of intervertebral discs is controversial. This study aimed to determine a possible relationship between bacterial and viral colonization and low-grade infection of the discs.

**Methods:**

We investigated 447 disc samples from 392 patients. Microbiological culture was used to examine the samples for bacterial growth, polymerase chain reaction (PCR) was used for detection of herpes simplex virus types 1 and 2 (HSV-1, HSV-2) and *Cytomegalovirus* (CMV), and histopathological analysis was used to detect signs of inflammation. The results were compared between subgroups organized according to gender, age, location of the samples, surgical approach, preoperative C-reactive protein (CRP), preoperative and 6 months postoperative Oswestry Disability Index (ODI) and Neck Disability Index (NDI), and Modic changes (MC) of the corresponding endplates. Also, we assessed the occurrence of postoperative infections within 6 months.

**Results:**

Microbiological culture was positive in 38.78% of the analyzed intervertebral discs. Altogether, 180 bacteria were isolated. Coagulase-negative staphylococci (CONS) (23.41%) and *Cutibacterium acnes* (18.05%) were the most frequently detected microorganisms. None of HSV-1, HSV-2, or CMV were detected. Male patients (p = 0.00036) and cervical segments (*p* = 0.00001) showed higher rates of positive culture results. Ventral surgical approaches ( *p* < 0.001) and Type 2 MC (*p* = 0.0127) were significantly associated with a positive microbiological result (* p*< 0.001). Neither pre- nor postoperative ODI and NDI are associated with positive culture results. In 4 (1.02%) patients, postoperative spondylodiscitis occurred.

**Conclusions:**

With 447 segments from 392 patients, we present one of the largest studies to date. While disc degeneration caused by HSV-1, HSV-2, and CMV seems unlikely, we found positive microbiological culture results in 38.78% of all discs. The role of local skin flora and sample contamination should be the focus of further investigations.

**Level of Evidence:**

III.

*Trial registration*: The study was registered at ClinicalTrials.gov (ID: NCT04712487, https://www.clinicaltrials.gov/ct2/show/study/NCT04712487).

**Supplementary Information:**

The online version contains supplementary material available at 10.1186/s10195-022-00633-y.

## Introduction

There has been long-lasting discussion on whether intervertebral disc degeneration and low back pain (LBP) are caused by low-grade infection of the discs [1–3]. In 2001 Stirling et al. [1] were the first to mention the association between low-grade infection of the intervertebral discs and sciatica. In 1977 Marshall et al. [4] reported elevated immunoglobulin levels in patients with sciatica. Based on these findings, Stirling et al. [1] examined disc tissue for the presence of microorganisms and found positive microbiological culture results in 53% of the samples. *Cutibacterium acnes* (formerly *Propionibacterium acnes*) and coagulase-negative staphylococci (CONS) are the most common microorganisms accused of causing low-grade infections [5–7]. *Cutibacterium acnes* is an anaerobic, gram-positive bacterium and part of the normal skin flora. Besides its role in acne, it can cause, as an opportunistic pathogen, postoperative and device-related infections [8]. CONS are gram-positive, coagulase-negative, facultative anaerobic cocci, which occur mainly in clusters and colonize human skin and mucous membranes. Furthermore, CONS represent one of the major nosocomial pathogens [9]. Besides bacteria, viruses have also been identified in intervertebral disc tissue [10]. Several studies have tried to figure out whether there is an association between low-grade infection and signs of inflammation on histopathological examination. However, none of them was able to find such an association [11, 12].

Microorganisms are also held responsible for Modic changes (MC) [13–15]. MC represent vertebral bone marrow lesions and are divided into three types. Type 1 (low signal on T1-weighted MRI and high signal on T2-weighted MRI) represent bone marrow edema and inflammation. Type 2 (high signal on T1- and T2-weighted MRI) represent the conversion of red bone marrow into yellow fat marrow. Type 3 (low signal on T1- and T2-weighted MRI) describe bony sclerosis [16, 17]. There are several hints in the literature that MC are associated with LBP [18, 19]. While high-grade spondylodiscitis usually shows type 1 MC combined with disc space signal alterations, low-grade infection might cause only MC without disc signal changes [20]. Furthermore, type 1 MC are significantly more likely to occur when the adjacent vertebral discs showed a positive culture result with anaerobic bacteria like *Propionibacterium acnes* [21].

Due to all these findings, antibiotic treatment in patients suffering from LBP and MC has been discussed in the literature [2, 22].

There is little literature concerning the influence of the level, kind of approach, and viral infections on the degeneration of the disc. The purpose of this prospective study was to evaluate the frequency of low-grade bacterial and viral infections of the intervertebral discs in an unselected, real-life patient population undergoing surgery for degenerative pathologies of the spine.

## Materials and methods

### Study design and patients

The study was designed as prospective, observational study. We collected disc samples intraoperatively in patients undergoing discectomy and fusion surgery of the entire spine due to degenerative pathologies between December 2018 and January 2020. Exclusion criteria were age < 18 years, acute spondylodiscitis (i.e., increased inflammatory parameters, fever, reduced general condition), tumor, and traumatic fractures.

The following epidemiological and clinical data were collected: gender, age, involved segments, surgical approach, preoperative C-reactive protein (CRP; reference range 0.0–0.5 mg/dl), and preoperative and six months postoperative Oswestry Disability Index (ODI) and Neck Disability Index (NDI), both expressed as percentages. Also, we assessed the occurrence of postoperative infections within six months after surgery. MC were assessed for each operated segment. In this study, only patients with a ventral surgical approach in the cervical region or a dorsal approach in the thoracic region were included. In the lumbar region, we chose a ventral approach for anterior lumbar interbody fusion (ALIF), a lateral approach for oblique or extreme lateral interbody fusion (OLIF, XLIF), and a dorsal approach in cases of standard microdiscectomy, posterior transforaminal interbody fusion (PLIF) or transforaminal interbody fusion (TLIF). The collected samples were sent to the Institute of Pathology and Microbiology for further examination using microbiological culture, polymerase chain reaction (PCR) and histopathological analysis.

### Collection of intraoperative samples

Each patient received a single shot of 1500 mg cefuroxime as a prophylaxis 30 min before surgery. In the case of penicillin or cephalosporin allergy, 600 mg clindamycin was administered. For skin disinfection, octenidine dihydrochloride and propanol was used. After sterile draping, skin incision was performed. Discectomy was performed and a sample of the vertebral disc was split into two parts. One part was sent unfixed for further microbiological and PCR examination, while the remaining tissue was placed in a vial containing a 4% solution of formaldehyde for histopathological analysis. If more than one spinal segment was operated on, tissue from each intervertebral disc was collected separately and assigned to the correct segment. Further analysis was then carried out separately for all affected segments.

### Microbiological culture

Tissue samples were mechanically homogenized and then plated onto Columbia blood agar, chocolate blood agar, and Schaedler agar. Sample processing and inoculation were conducted in a laminar flow workbench. The samples were incubated at 36 °C under aerobic (Columbia agar), 5% CO_2_ (Chocolate agar) or anaerobic (Schaedler agar) conditions and assessed for microbial growth daily.

In cases of growth, identification of microorganisms was performed using the Vitek 2 (Biomerieux) and the Maldi TOF (Bruker) systems. Final diagnosis of bacterial growth was made after 7 days of incubation.

### Real-time PCR

Fresh tissue samples were cut into small fragments under a sterile workbench (Holten Lamina Air). Pieces with a total volume of about 3 × 3 × 3 mm were transferred to a sterile 2 ml microcentrifuge tube containing 800 μl of ATL buffer (Qiagen, Germany) and 100 μl of proteinase K (20 mg/ml, Qiagen) to undergo a proteinase K digestion at 56 °C for a minimum of 2 h (maximum overnight). DNA was extracted using the QIAsymphony instrument (Qiagen) in combination with the QIAsymphony DSP Virus/Pathogen Mini Kit (Qiagen), as described in the manufacturer’s instructions. The DNA concentration was measured spectrophotometrically using a Nanodrop 1000 (ThermoFisher). A maximum of 100 ng/µl DNA was used for PCR. Real-time PCR for *Cytomegalovirus* (CMV) and herpes simplex virus types 1 and 2 (HSV-1, HSV-2), including an extraction control (inhouse method), was performed using primers and probes (TIB Molbiol, Table 1) and a 2 × TaqMan Fast Universal PCR Master Mix (ThermoFisher). Real-time PCR amplification was done using the AB 7500 Fast DX Real Time System (ThermoFisher) with a thermal cycling profile of 95 °C for 2 min followed by 50 cycles of 95 °C for 5 s, 60 °C for 35 s and 72 °C for 2 min. Another inhouse real-time PCR was performed to assess specimen quality and DNA integrity with a 300 bp housekeeping gene (PLZF, TIB Molbiol, Table 1). A melting curve analysis was performed using 2 × Power Up SYBR Green Master Mix (ThermoFisher). For all analyses, the ABI 7500 documentation software (SDS v1.4.1 and 7500 Software v2.0.6, ThermoFisher) was used.

**Table 1:** Primer and TaqMan probes used for detection of HSV-1, HSV-2, and CMV

Primer	Sequence	Amount
HSV forward	GAGTGCGAAAA(A/G)ACGTTC	10.0 pmol
HSV reverse	GTCTACGAGGACGGCC	10.0 pmol
CMV forward	GACACAACACCGTAAAGC	8.0 pmol
CMV reverse	CAGCGTTCGTGTTTCC	8.0 pmol
PLZF/X1U	GCGATGTGGTCATCATGGTG	1.6 pmol
PLZF/X1L	CGTGTCATTGTCGTCTGAGGC	1.6 pmol
TaqMan probe		
HSV-1	CGT CAT CTA CGG GGG TAA GAT GC	4.0 pmol
HSV-2	TCA TCT GCG GGG GCA AGA T	4.0 pmol
CMV	TCC TCG CAG AAG GAC TCC AG	2.0 pmol
Extraction control	AGC CGG ATC AAG CGT ATG C	1.0 pmol

### Histopathological analysis

Tissue for histopathological analysis was placed in a vial containing a 4% solution of formaldehyde. The vial was forwarded to the laboratory of neuropathology where the tissue was placed in Osteomoll (Merck, Germany, HC91222036, 1.01736.1000), a rapid decalcifier solution for histology containing hydrochloric acid and formaldehyde, for 2 h. The tissue was placed in the tissue processor overnight and subsequently embedded in paraffin. Sections 5 µm thick were cut, stained with hematoxylin and eosin and cover-slipped. Retrospectively, additional sections were cut and stained for the demonstration of various infectious agents. The histopathological examination of the tissue included evaluation of the presence of areas with necrosis and clusters of large reactive chondrocytes as signs of tissue regeneration. The degree of degeneration was rated as light, moderate or severe.

### Statistical analysis

Nominal variables are described with absolute and relative frequencies. Metric variables are reported with the corresponding mean and standard deviation (SD). Fisher’s exact test was used to test for associations between nominal variables. Wilcoxon-Mann–Whitney tests were performed to test for differences between two groups of non-normal metric or ordinal variables. Logistic regression analysis was conducted to model the risk of a positive microbiological culture and the risk of Modic signs using a set of explanatory variables. Although multiple observations were collected for several patients, this analysis did not adjust for such multiplicity and assumes that all observations are independent. Regression models for positive culture results and all three types of Modic signs were fitted separately for the subgroups of patient samples from the cervical spine and from the thoracolumbar spine. Variables were selected stepwise using the Akaike Information Criterion as a quality measure for the selection procedure. The level of significance was set to 0.05.

## Results

We investigated 447 samples from 392 patients (189 female and 203 male) with a mean age of 58.1 years. Baseline patient characteristics are shown in Table 2. 93 (23.7%) patients underwent cervical, 4 (1.02%) thoracic, and 295 (75.3%) lumbar spine surgery with discectomy. In most patients (262, 66.84%) we used a dorsal surgical approach, followed by ventral (117, 29.85%) and lateral procedures (13, 3.32%). In the cervical spine we used only a ventral approach. In 24 patients, a ventral approach was used for ALIF surgery. In 13 patients, we performed a lateral approach for fusion surgery by OLIF or XLIF. 161 patients underwent discectomy with fusion. Thirty-six patients had two segments, eight had three segments, and one patient had four segments operated on. Altogether, we examined 447 segments and disc samples. The most frequent lumbar segment was L4/5 (29.37%). The most frequent cervical segment was C5/6 (10.54%).

**Table 2:** Baseline patient characteristics

	n	%	
Total number of patients	392	100	
Cervical spine	93	23.7	
Thoracic spine	4	1.02	
Lumbar spine	295	75.3	
Dorsal	262	66.84	
Ventral	117	29.85	
Lateral	13	3.32	
1-segment surgery	347	88.52	
2-segment surgery	36	9.18	
3-segment surgery	8	2.04	
4-segment surgery	1	0.26	
	male	female	
Gender	203 (51.79%)	189 (48.21%)	
	Mean	SD	Median
Age (years)	58.09	14.83	58.88
ODI (%)	56.52	16.82	58
NDI (%)	39.87	17.28	38.05
CRP (mg/dl)	0.4	0.63	0.22

Mean, standard deviation and median for metric variables, and absolute and relative frequencies for nominal and ordinal variables for the total study population are provided

**Table 3:** Number and frequency of all isolated microorganisms

	N	Frequency of isolation (%)	Proportion of all microbes isolated (%)
Total positive culture results	180	43.9	100
*Cutibacterium acnes*	74	18.05	41.1
Coagulase-negative staphylococci	96	23.41	53.3
*Staph. epidermidis*	75	18.29	41.7
* Staph. saccharolyticus*	8	1.95	4.4
*Staph. warneri*	4	0.98	2.2
*Staph. capitis*	6	1.46	3.3
*Staph. hominis*	2	0.49	1.1
*Staph. lugdunensis*	1	0.24	0.5
*Corynebacterium* spp.	2	0.49	1.1
Alpha-hemolytic streptococci	2	0.49	1.1
*Strept. parasanguinis*	1	0.24	0.5
*Strept. mitis*	1	0.24	0.5
*Bacillus cereus*	2	0.49	1.1
*Staph. aureus*	1	0.24	0.5
*Granulicatella adiacens*	1	0.24	0.5
*Paenibacillus* spp.	1	0.24	0.5
*Lactobacillus* spp.	1	0.24	0.5

### Microbiological culture

Culture results were available for 410 segments. We saw a positive result in 159 (38.78%) segments. A total of 180 microbes were isolated in these 159 segments. The number and frequency of all found microorganisms are provided in Table 3. CONS were the most frequently identified microorganisms, found in 96 segments (23.41%), followed by *Cutibacterium acnes*, found in 74 segments (18.05%). Male patients were significantly more frequently affected (*p* = 0.00036).

We found at least one positive culture result in 88 (48.35%) of the male patients, but in only 53 (29.76%) female patients. Cervical segments (57.14%) showed a positive culture result significantly more often than lumbar segments (32.08%) (*p* < 0.001). A test for significance was not performed for thoracic segments due to the low case number (Table 2; n = 4).

Logistic regression was performed separately for cervical and lumbar segments to reveal parameters that influence the incidence of positive microbiological culture. Included parameters were gender, age, CRP, ODI for lumbar and NDI for cervical segments, surgical approach, and signs of inflammation in histopathological analysis. Females showed a significantly lower risk of having a positive culture result in the cervical spine (*p* < 0.001). Age (*p* = 0.133), CRP (*p* = 0.982) and NDI (*p* = 0.936) did not show significant effects on the incidence of possible positive culture results. In the lumbar spine, no parameters had a significant effect on the frequency of positive culture results (age *p* = 0.112; gender *p* = 0.182; lateral approach *p* = 0.412; ventral approach *p* = 0.594; CRP *p* = 0.498; ODI *p* = 0.225; signs of inflammation in histopathology *p* = 0.778). Detailed results of the logistic regression model for microbiological culture can be found in the Additional file 1: Tables S4–S6.

### PCR for viral infection

PCR was performed in all 447 segments. We could not detect any infection by HSV-1, HSV-2 or CMV.

### Histopathological examination

Results were available for 443 of the 447 segments. Analysis revealed signs of inflammation in 15 out of 443 (3.39%) disc samples. Eight samples showed light, five moderate, and two severe signs of associated degeneration. These positive results were found in patients who underwent dorsal surgery in the lumbar spine for discectomy alone. Three of the segments with histological signs of inflammation showed positive type 2 MC. In the logistic regression analysis, inflammatory signs were not significantly associated with a positive culture result (p = 0.778).

### Surgical approach

Sixty-one (55.45%) of the 110 patients operated on with a ventral approach had at least one positive culture result. Seventy-three (30.80%) of the 237 patients operated on with a dorsal approach showed a positive result, and 7 of 13 (53.85%) patients operated on with a lateral approach were positive. Ventral approaches were significantly associated with a positive result (*p* < 0.001). Segments where a dorsal approach was used showed a positive culture significantly less frequently (*p* < 0.001). We found no significant association (*p* = 0.386) in the lateral approach group.

### Preoperative parameters

Mean CRP was 0.39 mg/dl (SD: 0.71) in patients with a positive culture result and 0.42 mg/dl (SD: 0.61) in patients with a negative culture result. The difference was not significant (*p* = 0.2162).

The mean ODI was 53.99% (SD: 17.82) in culture positive patients and 58.14% (SD: 16.01) in culture negative patients (*p* = 0.1193). Mean NDI was 38.3% (SD: 17.49) in culture positive patients and 43.13% (SD: 17.23) in culture negative patients (*p* = 0.2257).

### Postoperative parameters

At 6 months postoperative, the mean ODI was 21.74% (SD: 22.02) in the culture-positive group and 29.18% (SD: 18.82) in the culture-negative group. With a p-value of 0.138, the Wilcoxon-Mann–Whitney test yields an insignificant difference. NDI 6 months postoperative was 23.54% (SD: 25.75) in the culture-positive, and 25.72% (SD: 21.23) in the culture-negative group (*p* = 0.5332). Postoperative infections occurred in 4 of 392 (1.02%) patients (one female, three male). All four were cases of spondylodiscitis in the segment in which surgery was performed. In two cases, additionally, a psoas abscess occurred. All patients were older than 60. Three of the patients showed a positive intraoperative culture of the disc tissue; one was positive for *Cutibacterium acnes*, one for CONS, and one for both *Cutibacterium acnes* and CONS. We were able to isolate microorganisms causing the postoperative infection in two of the four cases, both from punctures of the psoas abscess. In one case, *Staph. aureus* was isolated, in the other case, *Cutibacterium acnes*. The patient in whom *Staph. aureus* was isolated postoperatively did not show any microorganisms in the intraoperative disc tissue culture. The patient in whom *Cutibacterium acnes* was isolated postoperatively did show CONS (*Staph. hominis*) in the intraoperative culture.

### Modic changes

MRI for analysis of MC was available for all 447 segments. We found 45 type 1 MC (10.07% of all segments), 118 type 2 MC (26.4%) and five type 3 MC (1.12%) on MRI. Microbiological culture was available in 410 segments. In these 410 segments, we found 39 type 1 MC, 113 type 2 MC and five type 3 MC. We saw a statistically significant relationship between type 2 MC and a positive microbiological result (*p* = 0.0127). In contrast, there were no statistically significant associations concerning type 1 MC and type 3 MC (*p* = 0.3052 and *p* = 0.0767 respectively). Logistic regression was performed separately for cervical and lumbar segments to reveal parameters that might influence the incidence of Modic signs. Evaluated parameters were age, gender, culture result, CRP, ODI, NDI, and surgical approach. The logistic regression model for cervical segments showed that gender was the only parameter to influence the occurrence of any MC significantly. Being female was associated with a significantly lower risk of type 2 MC (*p* = 0.021). In the lumbar spine, the only significant parameter to influence the appearance of MC was the ODI. A higher ODI decreased the risk of type 2 MC significantly (*p* = 0.009). Detailed results of the logistic regression model for Modic changes are provided in the Additional file 1: Tables S7–S11.

## Discussion

We saw growth of at least one bacterium in 38.78% of all examined discs. This is similar to the rates reported in the literature [6, 11]. Iyer et al. [20], reviewing the results of 14 studies with 1,454 patients, found an average frequency of bacterial growth of 41.2%, with growth of *Cutibacterium acnes* in 28.7% and of CONS in 9.8% of all examined discs. *Cutibacterium acnes* was the most frequently isolated organism, responsible for 73.9% of all positive findings, followed by CONS, which accounted for 25.5% of all findings. Alpha-hemolytic streptococci, *Micrococcus* spp., *Corynebacterium* spp., *Actinomyces* spp., and *Neisseria* spp. were detected in 1% or fewer of all cultures. Interestingly, while many studies found higher rates of *Cutibacterium acnes* than CONS [1, 5, 6, 21, 23–26], CONS were the most frequently isolated organisms in our study. *Cutibacterium acnes* is associated with hair follicles and pilosebaceous units [27–30]. Preoperative disinfection insufficiently penetrates the follicles, where approximately 25% of the cutaneous bacterial population is localized [31]. Presumably, this could lead to a higher chance of survival by *Cutibacterium acnes* than by the more superficially located CONS, which would explain the proportionally higher rates of *Cutibacterium acnes* findings in the literature. On the other hand, the more superficial location of CONS on the skin might facilitate contamination of the samples by CONS. Furthermore, CONS belong to the bacterial ecosystem of hospitals, public transport, and rural homes [32], which might also increase the risk of contamination.

Male participants, cervical segments and an anterior approach were significantly associated with a higher rate of positive culture results. Contrarily, previous studies found an equal distribution of *Cutibacterium acnes* findings in the cervical and lumbar spine [11, 33]. Chen et al. [34] examined only cervical discs and reported a positive culture rate of 13.6%, which is distinctly lower than our rate. Only few studies evaluated the influence of the surgical approach and level of the operated level on the rate of positive cultures. *Cutibacterium acnes* and staphylococci colonize sebaceous, gland-rich sites such as the face, scalp, chest, and back. They are also present at dry and moist skin sites such as buttocks, forearm, inner elbow, and umbilicus [27, 35]. Furthermore, men are colonized by *Cutibacterium acnes* more often than women [36]. For future studies, further investigations into the role of the skin flora and its association with positive microbiological culture results from intervertebral discs would be of interest.

We evaluated CRP, ODI, and NDI as preoperative clinical predictors for positive culture results. A slightly elevated CRP value in particular, could indicate a low-grade infection and the need for antibiotic therapy. However, we could not detect any significant effect. In fact, mean preoperative CRP, as well as mean ODI and NDI, were slightly lower for patients with positive culture results, although the difference between the positive and negative culture groups were not significant (CRP *p* = 0.2162; ODI *p* = 0.1193; NDI *p* = 0.2257).

Considering the postoperative outcome associated with the microbiological culture, neither ODI nor NDI were significantly associated with the culture result. Three of four patients, who developed a postoperative infection, showed a positive result from the interoperative microbiological culture. However, in the two patients in whom a microorganism responsible for the postoperative infection could be isolated, the bacteria causing the postoperative infection were different from the microorganisms detected in the disc culture. The clinical relevance the intraoperative disc culture for prediction and treatment of postoperative spondylodiscitis, therefore, remains unclear and doubtful.

Studies on viral infections of the intervertebral disc are rare [10, 37, 38]. Alpantaki et al. [10] reported a prevalence of positive PCR tests of 56.25% for HSV-1 and 37.5% for CMV in 16 patients. Walker et al. [38] investigated herniated disc tissue of 15 patients for herpesvirus DNA and did not detect any viral pathogens. In our sample, we found no evidence of any infection of the intervertebral discs by HSV-1, HSV-2, or CMV. With 447 examined discs, we present the biggest study to date investigating this topic. Considering our results, an association between HSV-1, HSV-2, and CMV infections and degeneration of the intervertebral discs seems highly unlikely.

In 3.39% of our disc samples, we saw histopathological signs of inflammation. The origin of all these specimens was lumbar disc herniation. We did not find a significant association between a positive culture result and histopathological changes. Rigal et al. [12] examined a possible association between type 1 MC and low-grade infection in the lumbar spine. They found no histopathological signs of inflammation. This is in line with Coscia et al. [11], who found positive cultures in 45% of discs (76 of 169) but no histological inflammation signs. The authors hypothesized that bacteria within the discs form a biofilm, allowing endemic colonization of the intervertebral discs without inflammation and degeneration. Capoor et al. confirmed the existence of biofilm formations using laser microscopy [7]. Nevertheless, they could not prove whether the bacteria's presence in the human disc is a physiological process or an infection.

According to the literature [21, 39], we expected type 1 MC to be significantly associated with the occurrence of bacteria. In our sample, type 2 MC were significantly related to positive microbiological cultures, while type 1 and type 3 MC were not. It must be mentioned, however, that type 1 and type 3 MC were much less frequently observed than type 2 MC. Dudli et al. [40] found that injection of *Cutibacterium acnes* into intervertebral discs induced type 1 MC in an animal model. Nevertheless, there is no consensus in the literature regarding MC and the administration of antibiotics. Albert et al. administered amoxicillin-clavulanate (500 mg/125 mg) tablets at 8-h intervals for 100 days to patients suffering from chronic LBP with type 1 MC [2]. This study's antibiotic protocol was significantly more effective than the placebo group in all outcomes. In contrast, Bråten et al. could not show any clinically important effect of three months of oral antibiotic treatment in patients with chronic LBP and MC [22]. We could not find an association between MC and a worse clinical status, as measured by ODI or NDI. Contrarily, we found that a higher ODI (i.e., a worse clinical status) decreases the frequency of the presence of type 2 MC significantly.

There are several limitations to our study. We collected only one sample of the disc. Due to the lack of more control cultures, contamination cannot be excluded. However, Capoor et al. [6] state that endogenous controls do not necessarily allow identification of contaminated disc tissue. A possible way to distinguish between skin contamination and disc infection could be comparative genomics of bacteria from the skin and disc tissue [6, 41]. Positive results of the microbiological culture could be affected by various parameters, such as operation time and different surgeons performing the procedures. Future studies should also aim to investigate the influence of these parameters on the rate of positive findings. Also, antibiotic prophylaxis could possibly lead to a higher rate of false negative culture findings [42]. However, we wanted to show the prevalence of Cutibacterium acnes and other pathogens in a real-life patient population. We believe that preoperative antibiotic prophylaxis is an indispensable part of routine spine surgery and should not be postponed until tissue samples are collected.

## Conclusion

To our knowledge, this study investigating disc samples from 447 vertebral segments is one of the largest to date. An association of disc degeneration and infections caused by HSV-1, HSV-2, and CMV seems to be unlikely. Histopathological inflammation signs in degenerated discs are rare and not associated with microbiological findings. In our cohort, an anterior approach, cervical spine surgery, and male patients had a significantly higher risk for positive microbiological culture results. There is a statistically significant association between type 2 MC and positive microbiological results. Notably, this relationship was clinically irrelevant. We could not find an association between pre-/postoperative ODI and NDI and culture results. With the administration of preoperative antibiotic prophylaxis and the lack of control cultures, an important limitation of our study is that false negative results with respect to culture contamination can not be ruled out. Further research on the clinical relevance of higher rates of positive microbiological results associated with the surgical approach, segment level, and gender should be conducted. When considering these factors, the role of local skin flora and sample contamination should be the focus of further investigations.

## Supplementary Information

**Additional file 1: Table S4.** Pseudo-R2 values of the regression models for positive microbiological culture. **Table S5**. Results of the logistic regression model for positive microbiological culture of cervical segments. **Table S6**. Results of the logistic regression model for positive microbiological culture of lumbar segments. **Table S7**. Pseudo-R2 values of the regression models for Modic signs. **Table S8**. Results of the logistic regression model for positive Modic changes type 1 for cervical segments. **Table S9**. Logistic regression model for positive Modic changes type 2 for cervical segments. **Table S10**. Results of the logistic regression model for positive Modic changes type 1 for thoracal and lumbar segments. **Table S11**. Results of the logistic regression model for positive Modic changes type 2 for thoracal and lumbar segments.

## Data Availability

The datasets used and/or analyzed during the current study are available from the corresponding author on reasonable request.

## Abbreviations

ALIF: Anterior lumbar interbody fusion
CMV: *Cytomegalovirus*
CONS: Coagulase-negative staphylococci
CRP: C-reactive protein
HSV-1: Herpes simplex virus type 1
HSV-2: Herpes simplex virus type 2
LBP: Low back pain
MC: Modic changes
NDI: Neck Disability Index
OLIF: Oblique lateral interbody fusion
PLIF: Posterior lumbar interbody fusion
TLIF: Transforaminal lumbar interbody fusion
XLIF: Extreme lateral interbody fusion
