# Supplementary material for: Frequency and characteristics of bacterial and viral low-grade infections of the intervertebral discs: a prospective, observational study
Source: J Orthop Traumatol. 2022 Mar 18;23:15. doi: 10.1186/s10195-022-00633-y (PMC8933588; doi:10.1186/s10195-022-00633-y)
Supplement: Supplementary file 1 — Additional file 1: Table S4. Pseudo-R2 values of the regression models for positive microbiological culture. Table S5. Results of the logistic regression model for positive microbiological culture of cervical segments. Table S6. Results of the logistic regression model for positive microbiological culture of lumbar segments. Table S7. Pseudo-R2 values of the regression models for Modic signs. Table S8. Results of the logistic regression model for positive Modic changes type 1 for cervical segments. Table S9. Logistic regression model for positive Modic changes type 2 for cervical segments. Table S10. Results of the logistic regression model for positive Modic changes type 1 for thoracal and lumbar segments. Table S11. Results of the logistic regression model for positive Modic changes type 2 for thoracal and lumbar segments. [file 10195_2022_633_MOESM1_ESM.docx]

|  | **CoxSnell** | **Nagelkerke** |
| --- | --- | --- |
| **Cervical spine** | 0.3191 | 0.4310 |
| **Lumbar spine** | 0.0448 | 0.0620 |

**Table 4: Pseudo-R^2^ values of the regression models for positive microbiological culture.**

|  | **Estimate** | **Exp(Estimate)** | **Std.Error** | **p-value** |
| --- | --- | --- | --- | --- |
| **(Intercept)** | 1.883 | 6.570 | 0.486 | 0.000 |
| **age** | 0.035 | 1.036 | 0.024 | 0.133 |
| **female gender** | -2.917 | 0.054 | 0.678 | 0.000 |
| **CRP** | 0.010 | 1.010 | 0.429 | 0.982 |
| **NDI** | -0.001 | 0.999 | 0.018 | 0.936 |

**Table 5: Results of the logistic regression model for positive microbiological culture of cervical segments.**Reference patient: 58 years old, male, preop. CRP 0.2, NDI 38

|  | **Estimate** | **Exp(Estimate)** | **Std.Error** | **p-value** |
| --- | --- | --- | --- | --- |
| **(Intercept)** | -0.0492 | 0.611 | 0.227 | 0.030 |
| **Age** | -0.016 | 0.984 | 0.010 | 0.122 |
| **Female gender** | -0.401 | 0.670 | 0.301 | 0.182 |
| **Lateral** | 0.531 | 1.700 | 0.647 | 0.412 |
| **Ventral** | 0.227 | 1.319 | 0.520 | 0.594 |
| **Thoracic spine** | -14.669 | 0.000 | 1021.134 | 0.989 |
| **CRP** | -0.227 | 0.797 | 0.335 | 0.498 |
| **ODI** | -0.011 | 0.989 | 0.009 | 0.225 |
| **Histopathology** | -0.174 | 0.840 | 0.618 | 0.778 |

**Table 6: Results of the logistic regression model for positive microbiological culture of lumbar segments.**

Reference patient: 58 years old, male, preop. CRP 0.2, ODI 38, dorsal approach, negative histopathology

|  | **CoxSnell** | **Nagelkerke** |
| --- | --- | --- |
| **Cervical spine (Modic 1)** | 0.0171 | 0.0413 |
| **Cervical Spine (Modic 2)** | 0.2089 | 0.3009 |
| **Thoracic and lumbar spine (Modic 1)** | 0.0588 | 0.1160 |
| **Thoracic and lumbar spine (Modic 2)** | 0.0440 | 0.0634 |

**Table 7. Pseudo-R^2^ values of the regression models for Modic signs.** Modic 3 was not included due to the low case number

|  | **Estimate** | **Exp(Estimate)** | **Std.Error** | **p-value** |
| --- | --- | --- | --- | --- |
| **(Intercept)** | -1.679 | 0.187 | 0.876 | 0.055 |
| **Age** | 0.015 | 1.015 | 0.031 | 0.619 |
| **Female gender** | -0.759 | 0.468 | 1.033 | 0.462 |
| **Positive microbiology** | -1.058 | 0.347 | 0.985 | 0.283 |
| **CRP** | 0.183 | 1.201 | 0.402 | 0.649 |
| **NDI** | 0.006 | 1.006 | 0.024 | 0.794 |

**Table 8. Results of the logistic regression model for positive Modic changes type 1 for cervical segments.**

Reference patient: 58 years old, male, negative microbiology culture, CRP 0.1, NDI 0.38

|  | **Estimate** | **Exp(Estimate)** | **Std.Error** | **p-value** |
| --- | --- | --- | --- | --- |
| **(Intercept)** | -1.046 | 0.351 | 0.686 | 0.127 |
| **Age** | 0.036 | 1.037 | 0.023 | 0.121 |
| **Female gender** | -1.545 | 0.213 | 0.738 | 0.036 |
| **Positive microbiology** | 1.094 | 2.987 | 0.696 | 0.116 |
| **CRP** | -0.778 | 0.459 | 0.810 | 0.337 |
| **NDI** | -0.013 | 0.987 | 0.016 | 0.436 |

**Table 9. Logistic regression model for positive Modic changes type 2 for cervical segments.**

Reference patient: 58 years old, male, negative microbiology culture, CRP 0.1, NDI 0.38

|  | **Estimate** | **Exp(Estimate)** | **Std.Error** | **p-value** |
| --- | --- | --- | --- | --- |
| **(Intercept)** | -2.283 | 0.102 | 0.397 | 0.000 |
| **Age** | -0.019 | 0.981 | 0.016 | 0.225 |
| **Female gender** | 0.452 | 1.571 | 0.450 | 0.315 |
| **Positive microbiology** | -0.736 | 0.479 | 0.523 | 0.160 |
| **Ventral** | 1.232 | 3.428 | 0.577 | 0.033 |
| **Lateral** | -0.522 | 0.593 | 1.082 | 0.630 |
| **CRP** | -0.461 | 0.631 | 0.478 | 0.335 |
| **ODI** | -0.023 | 0.977 | 0.014 | 0.088 |

**Table 10. Results of the logistic regression model for positive Modic changes type 1 for thoracal and lumbar segments.**

Reference patient: 58 years old, male, lumbar spine surgery, dorsal approach, negative microbiological culture, CRP 0.2, ODI 58, negative histopathology

|  | **Estimate** | **Exp(Estimate)** | **Std.Error** | **p-value** |
| --- | --- | --- | --- | --- |
| **(Intercept)** | -0.836 | 0.433 | 0.264 | 0.002 |
| **Age** | -0.008 | 0.992 | 0.011 | 0.435 |
| **Female gender** | -0.205 | 0.815 | 0.306 | 0.504 |
| **Positive microbiology** | 0.004 | 1.004 | 0.322 | 0.990 |
| **Ventral** | -0.214 | 0.808 | 0.548 | 0.697 |
| **Lateral** | -0.129 | 0.879 | 0.574 | 0.822 |
| **CRP** | -0.070 | 0.933 | 0.328 | 0.831 |
| **ODI** | -0.022 | 0.979 | 0.009 | 0.022 |
| **Histopathology** | -0.307 | 0.735 | 0.678 | 0.650 |

**Table 11. Results of the logistic regression model for positive Modic changes type 2 for thoracal and lumbar segments.**

Reference patient: 58 years old, male, lumbar spine surgery, dorsal approach, negative microbiological culture, CRP 0.2, ODI 58, negative histopathology
